# Bromide of Potassium for the Troubles of Teething

**Published:** 1869-08

**Authors:** A. A. Hoehling

**Affiliations:** Sur. U. S. Navy


Bromide of Potassium for the Troubles of Teething. By A. A. Hoehling, M. D., Sur. U. S. Navy.—The gratifying results which followed the use of the bromide of potassium in the only three cases of difficult dentition which have come under my care lately, must be my excuse for trespassing upon your valuable space. An additional reason for my communication, after such a limited experience, is that my return to naval duty renders it improbable that I shall myself have an early opportunity of fully testing the great efficacy which I believe this remedy to possess.

The first case was that of an infant aged 11 months, which had been brought up by hand. Its stomach had always been very irritable, and its nutrition was poor. Some three weeks before I saw the child, severe symptoms set in from teething; the gums had been lanced, and the child was nourished with great difficulty. It had been given paregoric and quinine injections, but without benefit. When I saw it there was little hope in my mind, for there were marked head symptoms, the cough so often met with in these cases, and debility. The stomach rejected much of the food which was given. I was convinced that the opiate was contraindicated on account of the brain symptoms, and tried hyoscyamus for a day or two. This did not answer, and I concluded to make use of the bromide. Doses of one grain every six hours were given, and immediately the child obtained rest; it did not roll its head about so much, and slept well. After a somewhat prolonged convalescence, during which the gums were freely lanced by the family physician, the child recovered. The previous condition of its stomach had probably much to do with its tedious convalescence.—The second case was in a child two years of age; he had a cough, and was somewhat listless; found him taking syrup ipecac. The gum over his lower molars was highly inflamed and puffy. Gave potassii bromidi gr. j. in a teaspoonful of aqua cinnamomi every four hours. In two days the child was well enough to dispense with farther attendance.—The third case was also a child two years of age, in which the molars were giving trouble. The child had an occasional chill, loss of appetite, bright red and swollen cheeks, profuse salivation. Gave the bromide, gr. j. ter die, and *immediate* relief was procured. His parents were then directed to give a dose of the medicine only when they should perceive that the child was in pain. When I last saw him he was not laboring under any symptoms indicative of difficult dentition.

In cases like the above we must relieve the pain, and all will go well; from my small experience I consider lancing the gums as a temporary expedient, and one which retards the appearance of the tooth. I believe that much of the irritation proceeds from the alveolar process itself, and is but moderately relieved by the hæmorrhage from an incision into the gum; the mere pressure upon the *gum* I do not regard as of the first importance. When the gum *is* lanced, it should be by two oblique incisions, if over a molar, cross cuts at right angles being useless, as the points of the crown are not set free. Opium is undoubtedly injurious in these cases, by increasing the tendency to brain congestion which already exists. We have then the bromide of potassium which tranquilizes the nervous system, relieves pain, and procures sleep; nature reinforced by such powerful aids soon accomplishes the rest for we have to do with a natural process, which requires but little help from us, if that little be well directed. The cough attendant upon difficult teething is undoubtedly well known to experienced practitioners, but yet it will do no harm to ask young beginners always to examine the gums in children with a cough, during the period of dentition, for it is not uncommon to see a child dosed with expectorants when attention to the gums would at once procure relief.

U. S. S. New Hampshire, Norfolk, Va.

